# Forearm Range of Motion in *Australovenator wintonensis* (Theropoda, Megaraptoridae)

**DOI:** 10.1371/journal.pone.0137709

**Published:** 2015-09-14

**Authors:** Matt A. White, Phil R. Bell, Alex G. Cook, David G. Barnes, Travis R. Tischler, Brant J. Bassam, David A. Elliott

**Affiliations:** 1 School of Engineering, The University of Newcastle, Callaghan, NSW 2308, Australia; 2 Australian Age of Dinosaurs Museum of Natural History, The Jump Up, Winton, Queensland, 4735, Australia; 3 School of Environmental and Rural Science, University of New England, Armidale, NSW 2351, Australia; 4 School of Earth Science, University of Queensland, St Lucia, Qld 4072, Australia; 5 Monash Biomedical Imaging, Monash University, Clayton, VIC 3168, Australia; 6 Monash e-Research Centre, Monash University, Clayton, VIC 3168, Australia; 7 Life Sciences Computation Centre, Parkville, VIC 3052, Australia; University of Utah, UNITED STATES

## Abstract

The hypertrophied manual claws and modified manus of megaraptoran theropods represent an unusual morphological adaptation among carnivorous dinosaurs. The skeleton of *Australovenator wintonensis* from the Cenomanian of Australia is among the most complete of any megaraptorid. It presents the opportunity to examine the range of motion of its forearm and the function of its highly modified manus. This provides the basis for behavioural inferences, and comparison with other Gondwanan theropod groups. Digital models created from computed tomography scans of the holotype reveal a humerus range of motion that is much greater than *Allosaurus*, *Acrocanthosaurus*, *Tyrannosaurus* but similar to that of the dromaeosaurid *Bambiraptor*. During flexion, the radius was forced distally by the radial condyle of the humerus. This movement is here suggested as a mechanism that forced a medial movement of the wrist. The antebrachium possessed a range of motion that was close to dromaeosaurids; however, the unguals were capable of hyper-extension, in particular manual phalanx I-2, which is a primitive range of motion characteristic seen in allosaurids and *Dilophosaurus*. During flexion, digits I and II slightly converge and diverge when extended which is accentuated by hyperextension of the digits in particular the unguals. We envision that prey was dispatched by its hands and feet with manual phalanx I-2 playing a dominant role. The range of motion analysis neither confirms nor refutes current phylogenetic hypotheses with regards to the placement of Megaraptoridae; however, we note *Australovenator* possessed, not only a similar forearm range of motion to some maniraptorans and basal coelurosaurs, but also similarities with Tetanurans (Allosauroids and *Dilophosaurus*).

## Introduction

**Fig 1:**
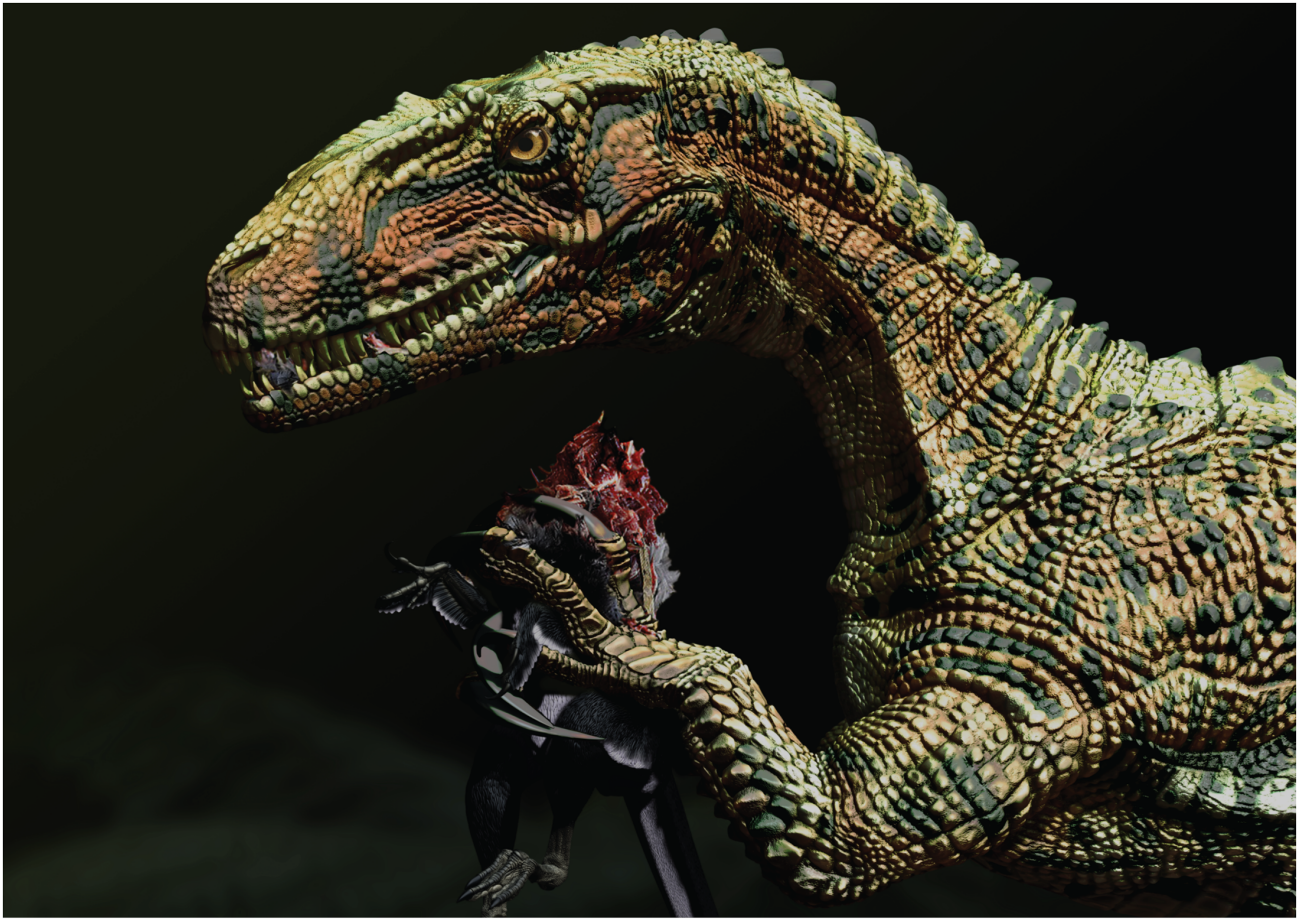
*Australovenator wintonensis*. A reconstruction of *Australovenator wintonensis* grasping a small theropod with its arms in a flexed posture.

*Australovenator wintonensis* Hocknull et al. [1] was a medium sized megaraptoran theropod that was discovered in Cenomanian-aged rocks near Winton, central Queensland, Australia (Fig 1) [2, 3]. Since the holotype description, additional skeletal elements pertaining to the holotype have been discovered following the ongoing preparation of concretions unearthed from the type locality [4, 5]. Hocknull et al. [1] originally recognised morphological similarities between *Australovenator*, Allosauroidea and Carcharodontosauria. However, subsequent workers place *Australovenator* in Megaraptoridae, which was established to represent the Gondwanan taxa, *Megaraptor*, *Orkoraptor* and *Aerosteon* although there is disagreement regarding the placement of Megaraptora within Tetanurae [6, 7]. A phylogenetic re-evaluation of *Australovenator* is still premature as a large quantity of material from the holotype locality is still undergoing preparation; however, the discovery of the nearly complete forearms permits examination of their potential range of motion (ROM) to infer their predatory function and provide additional insights into its evolutionary context. Forearm ROM studies in other theropods have been limited primarily because specimens infrequently preserve entire forelimbs. Nevertheless, comparable forelimb ROM analyses exist for representatives of Abelisauridae (*Carnotaurus sastrei* Bonaparte [8]) [9]; Alvarezsauridae (*Mononykus olecranus* Perle et al. [10]) [11]; Carcharodontosauridae (*Acrocanthosaurus atokenesis* Stovall and Langston [12]) [13]; the basal tetanuran (*Dilophosaurus wetherilli* Welles [14]) [15]; Dromaeosauridae (*Deinonychus antirrhopus* Ostrom [16] and *Bambiraptor feinbergi* Burnham et al. [17]) [18]; Ornithomimidae (*Ornitholestes hermanni* Osborn [19]) [20]; (*Struthiomimus altus* Lambe [21]) [22] and Tyrannosauridae (*Tyrannosaurus rex* Osborn [23]) [24]. Additionally, in some cases the ROM has been calculated based off figures cited by later ROM analysis (i.e ROM results of *Dilophosaurus* obtained from Fig 29 in Welles [14]) for ROM analysis in Senter and Parrish [15]. Due to the uncertain shape and extent of unpreserved soft tissues (e.g. enthesial cartilage), ROM has been commonly determined without allowances of these structures [13, 19, 24, 25, 26, 27]. However, there have been developments in understanding to what extent cartilage influenced limb lengths and how the joints functioned with the addition of cartilage [28–30]. Unfortunately, these methods do not accurately determine to what extent the morphology of the cartilage varied from the underlying bone. Additionally it was revealed that in some living archosaurs (e.g. *Alligator mississippiensis*, *Struthio camelus*) significant morphological variation exists between cartilaginous epiphyses and the underlying bone [29]. In the absence of prior knowledge of these variations in dinosaurs, accurate reconstructions of cartilaginous epiphyses remains indeterminate, especially considering ROM analysis. Subsequently, traditional bone-on-bone analysis remains the best method for investigating potential ROM. Recent studies focused on the ROM of crocodile shoulder joints [31] and wrist folding [32], have endeavoured to determine how soft tissues affect the ROM. These results show that bone-on-bone ROM is reasonably accurate however the results are more conservative. Soft tissue and bone spacing was discovered to increase the ROM, therefore potential ROM in dinosaurs and other fossil archosaurs are likely to be underestimated using a bone-on-bone approach [32]. Nevertheless, bone on bone models have shown that the manual digits of coelurosaurian theropods become less flexible compared to more basal taxa in exchange for increased ROM in the more proximal joints (elbow and shoulder). This tendency towards increased lateral extension (elevation) and retraction of the shoulder joint has also been implicated in the evolution of flight [13, 33].

Herein we provide the first bone-on-bone ROM estimates for a megaraptorid using the forearm elements of *Australovenator*. Our results are compared to the limited pre-existing dataset of theropod ROM studies in an attempt to identify evolutionary shifts in forelimb function and to infer potential behaviour.

## Materials and Methods

**Fig 2:**
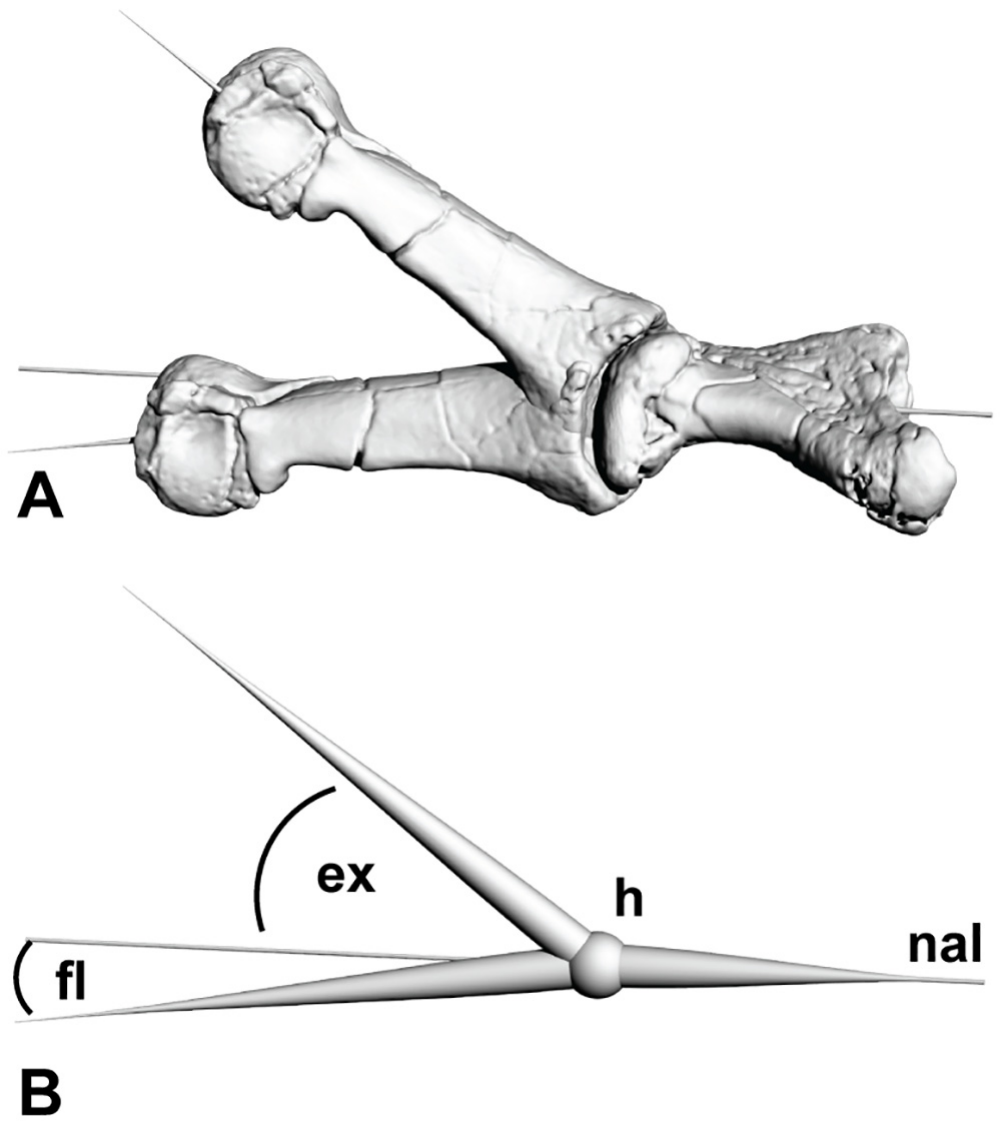
Determining range of motion from three dimensional meshes. (A) An example of determining the ROM of manual phalanx I-1 around metacarpal I in extended and flexed positions. The guide meshes are visible projecting from the specimen meshes. (B) The specimen meshes removed leaving the created guides used for determining the range of motion. A line of neutral articulation was created along a horizontal plain which is used as the basal limit for extension and flexion measurements. Abbreviations: ex extension angle; fl flexion angle; h hinge joint; nal neutral articulation line.

The ROM analysis comprised of a combination of holotype specimen articulation alongside digital articulation. To replicate three dimensional meshes of the holotype specimens, all of the forelimb elements were computed tomography (CT) scanned at Queensland X-ray, Mackay Mater Hospital in central eastern Queensland using a Philips Brilliance CT 64-slice machine producing 0.9mm slices. Mimics version 10.01 (Materialise HQ, Leuven, Belgium) was used to create three-dimensional meshes of specimens from the CT scans. These meshes were imported into Rhinoceros 5.0 (Robert McNeel and Associates, California USA); firstly to mirror image the left specimens where the right specimens were either too poorly preserved or weren’t discovered; secondly to convert the files into OBJ geometry definition files. The OBJ files were required for the creation of three dimensional portable document format (PDF) files and to import into the graphic design package Zbrush (Pixologic Inc, California, USA). Three dimensional ROM analysis ROM was achieved by both physically manipulating the holotype specimens and digitally orientating the meshes to their ROM limitations. These limitations were determined by the articular surface of the distal bone will reach but not move past the articular surface of the proximal bone [9, 11, 13, 15, 18, 25, 26, 34]. Zbrush was used to rearticulate the digital specimens to their ROM limits. The benefits of the digital articulation enabled all of the specimens to be articulated and viewed simultaneously in both extended and flexed views. To determine the extension and flexion limits of the metacarpals and phalanges a neutral horizontal articulation was established and referred to as zero degrees of rotation. ROM guides were drawn in Zbrush to analyse the ROM angles. Initially a sphere was drawn in the hinge joint acting as the central articulation point. Additional spheres were drawn as attachments to the initial anchored hinge sphere. The anchored spheres were projected to dissect the articular surfaces centrally. The spheres were converted to poly meshes within Zbrush and exported as OBJ geometry definition files to be viewed in Rhinoceros 5.0. The measuring capabilities of Rhinoceros enables the angle of the poly meshes to be determined by using the angle function under the ‘analyze’ menu (Fig 2).

In order to better understand the ROM of the antebrachium and bone spacing created by *in situ* cartilage we submitted a *Gallus* (common chicken) wing to both MRI and CT scans. Movement was achieved within the MRI scanner by securing the fleshed humerus to a board. A wooden shaft was strapped to the carpometacarpus in order extend and flex the forearm. A series of images were captured whilst the wing was being manipulated into extended and flexed positions. These images were combined into 3D meshes using Mimics 10.01 and imported into Rhinoceros to be viewed. The combined use of these three programs enabled accurate ROM analysis to be achieved and viewed in three dimensions.

### Specimens

The holotype specimen AODF85 was used for the ROM analysis which include humeri (S1 Fig), ulna (S2 Fig), radius (S3 Fig), radiale (S4 Fig), right distal carpal 1 (S5 Fig), metacarpal 1 (S6 Fig), manual phalanx I-1 (S7 Fig), manual phalanx I-2 (S8 Fig), metacarpal II (S9 Fig), manual phalanx II-1 (S10 Fig), manual phalanx II-2 (S11 Fig), manual phalanx II-3 (S12 Fig); manual phalanx III-3 (S13 Fig); manual phalanx III-4 (S14 Fig) [4]. These elements have been described in detail previously [1, 4] and readers are referred to these sources for full osteological descriptions.

Missing elements include the ulnare, metacarpal and phalanges of digit III, and the pectoral girdle. Due to these missing elements the ROM of the humerus and digit III could not be analysed.

### Three dimensional figures

Individual meshes of fossil specimens were loaded into a custom program that loads an Alias Wavefront (.obj) format mesh and compresses it into the Product Representation Compact (PRC) format, suitable for embedding in a PDF file as an interactive, 3-dimensional figure. We used a modified version of the program xrw2pdf from the S2VOLSURF tools [35], based on the S2PLOT programming library [36, 37]. PRC files were embedded in PDF documents as interactive figures using the LaTeX document preparation system, the movie 15 style file for LaTex supporting multimedia enhancements to PDF documents, and the JavaScript file s2plot-prc.js included with S2PLOT. When viewed in Adobe Reader or Adobe Acrobat (Adobe Systems Inc, California USA) on desktop computer systems, the resultant supplementary 3D figures enable the interactive rotation, zooming, and relighting of the fossil meshes.

### Ethics statement

All necessary permits were obtained for the described study, which complied with all relevant regulations. Permission to excavate the specimens from Elderslie station was obtained from the landholders who donate all specimens to the Australian Age of Dinosaur Museum of Natural History (AAOD). During excavation each specimen is given a preliminary field number for location and storing purposes. All specimens pertaining to the holotype *Australovenator wintonensis* are allocated the specimen number AODF604. The specimens are stored in a climate controlled type room at the Australian Age of Dinosaurs Museum 15km east of Winton, Queensland, Australia.

## Results

### Upper Arm

The pectoral girdle is unknown in *Australovenator* therefore we could not accurately model the ROM of the humeral joint. Despite this, it is possible to make some generalisations on the potential ROM based on the morphology of the humerus. The humeral head is more extensive caudally than cranially suggesting that it could have been extended to a sub-horizontal position but not retracted beyond a sub-vertical position [13]. This condition is consistent with most theropod groups except for advanced maniraptorans in which the scapular lies in a dorsolateral position relative to the ribcage and the glenoid faces laterally. Such an orientation allows the humerus to elevate past the sub-horizontal, consistent with the avian recovery stroke [13, 14, 20, 25, 38]. Deinonychosaurs have been posited as the immediate ancestors of birds [38] however, Senter [38] noted that their scapula and glenoid are oriented in the typical dinosaur fashion whereby the scapular blades are widely separated from the vertebrate column and lie laterally to the rib cage.

**Fig 3:**
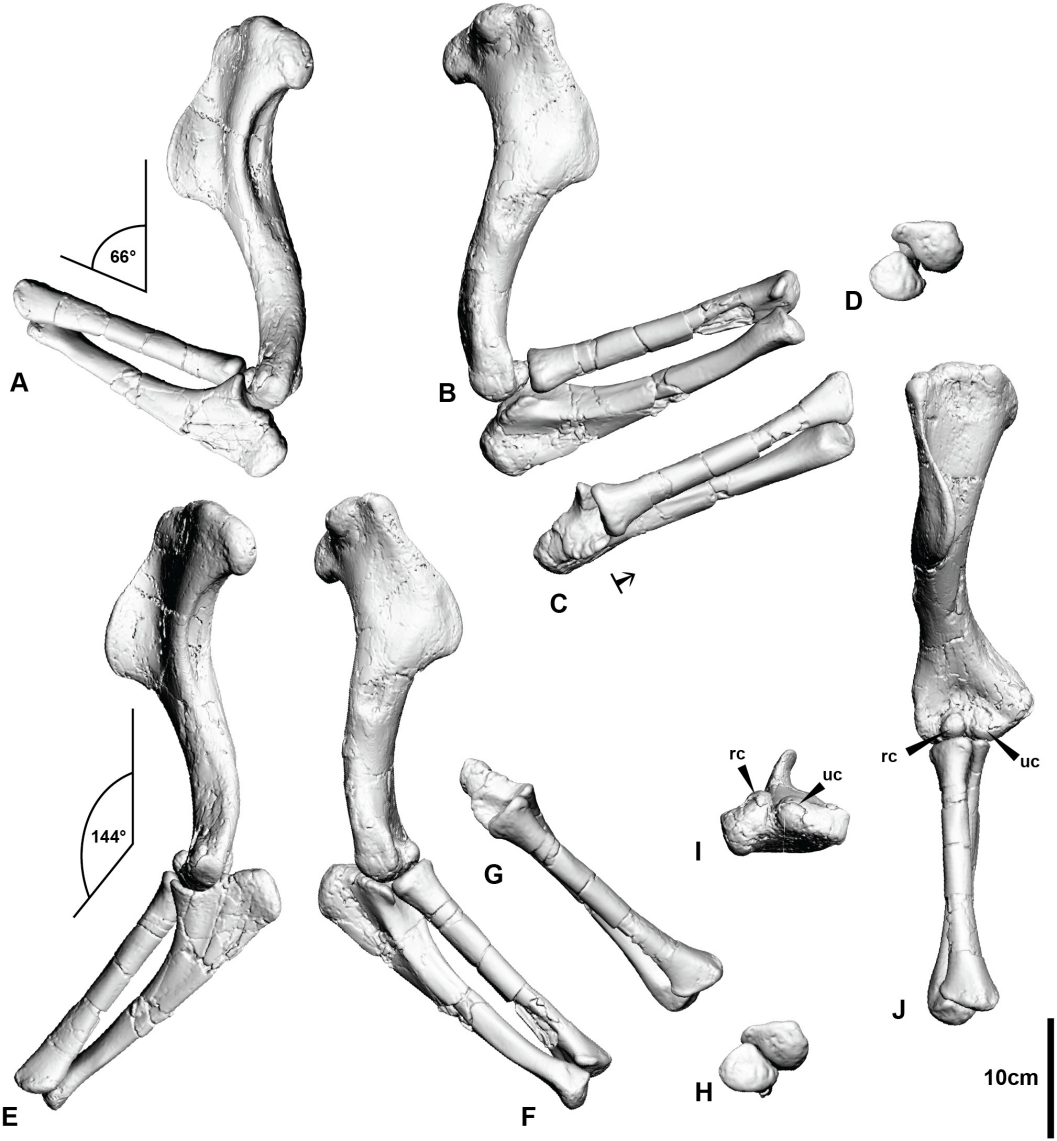
Articulated right humerus and antebrachium. (A) Right humerus and antebrachium flexed in medial view. (B) Right humerus and antebrachium flexed in lateral view. (C) Cranial view of radius and ulna in flexion displaying the distal movement of the radius indicated by the black arrow. (D) Distal articulation of the ulna and radius during flexion. (E) Right humerus and antebrachium extended in medial view. (F) Right humerus and antebrachium extended in lateral view. (G) Cranial view of radius and ulna in extended position with no distal displacement of the radius. (H) Distal articulation of the ulna and radius during extension. (I) Distal view of the humerus displaying the radial and ulna condyles. (J) Cranial view of the right humerus and antebrachium in extension displaying the position of the radial condyle which forces the distal displacement during flexion. Abbreviations: radial condyle (rc), ulna condyle (uc).

This orientation positions the glenoids ventrally, which prevents the humerus from being able to extend past the sub-horizontal [38]. Conversely, the enlarged deltopectoral crest likely permitted powerful retraction of the entire forelimb. The antebrachium was capable of swinging through an arc of 78° (maximum extension of 144°, maximum flexion of 66°; Fig 3, S15 Fig). A similarly high elbow ROM occurs in basal Maniraptoriformes (95° in *Ornitholestes* [20] and dromaeosaurids 68° in *Bambiraptor* and 99° in *Deinonychus* [18].

**Fig 4:**
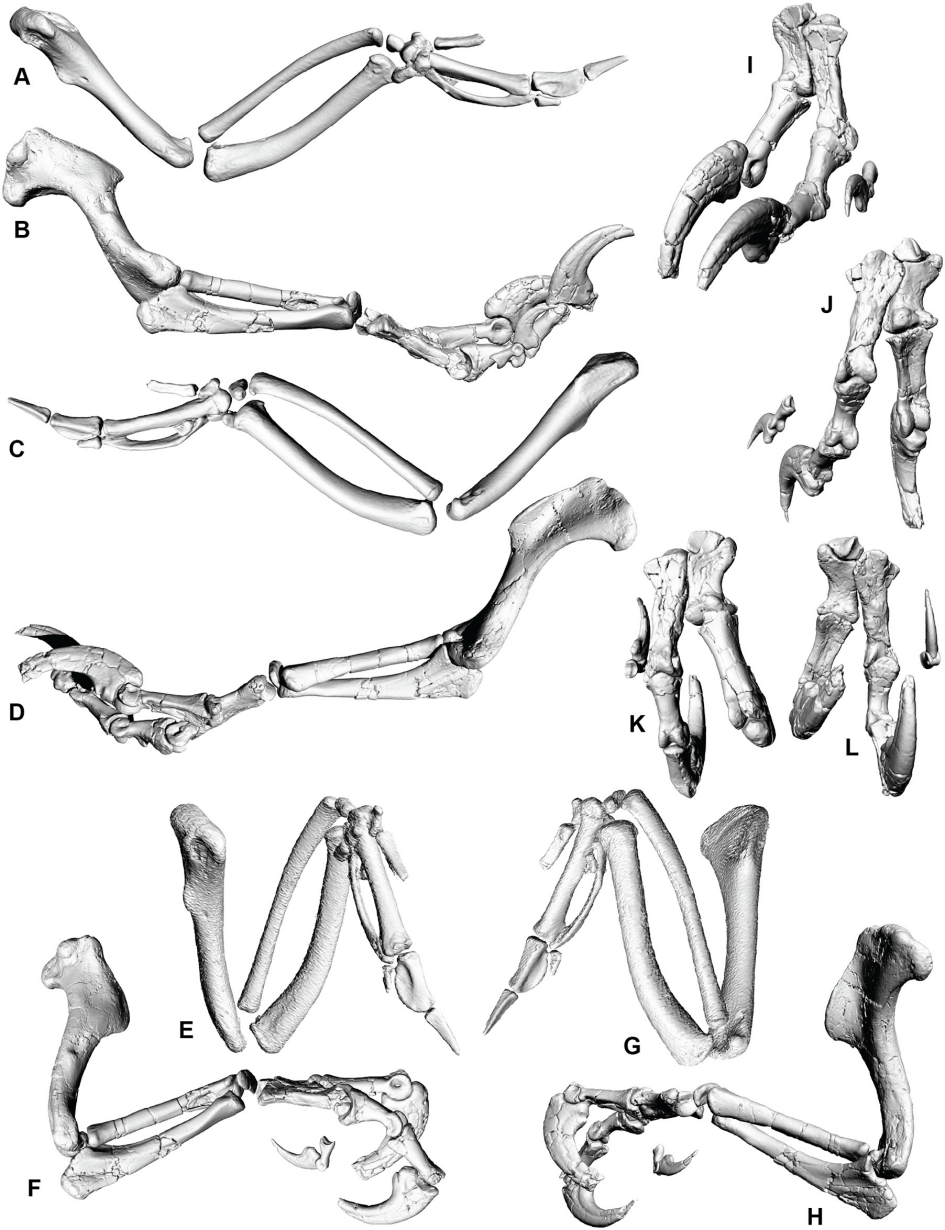
Right forearm and manus of *Australovenator* and *Gallus* forearm. Right forearm in extended lateral view; (A) *Gallus*. (B) *Australovenator*. Right forearm extended in lateral view; (C) *Gallus*. (D) *Australovenator*. Right forearm flexed in lateral view; (E) *Gallus*. (F) *Australovenator*. Right forearm flexed in medial view; (G) *Gallus*. (H) *Australovenator*. *Australovenator* manus; (I) extended cranial view; (J) extended ventral view; (K) flexed cranial view; (L) flexed ventral view.

The distal end of the humerus has a pronounced radial condyle and a smaller ulna condyle separated by a distinct intercondylar groove [4]. This groove received the lateral portion of the humeral facet of the ulna and dictated the flexion limit of the forearm. The surfaces where the radius and ulna articulate with each other are smooth and portray the ability of the radius to move distally and proximally during forearm flexion and extension (Fig 3). The pronounced radial condyle of the humerus forces the radius to move distally (12.2 mm) in relation to the ulna during flexion. This movement of the radius during forearm flexion is typical of many maniraptorans and extant birds [39]. In modern birds, the radius and ulna lie parallel to one another in the antebrachium when the wing is extended (e.g. for flight). During flexion (wing folding), however, the radius slides along a plane parallel to the ulna and forced distally by the radial condyle [38, 39, 40, 41]. This distal movement pushes the radiale into the carpometacarpus, which, in turn assists in the folding of the wing (Fig 4). Conversely, in *Australovenator* this movement appears to have created a medial movement of the wrist during flexion and would have been further accentuated by the presence of articular cartilage.

### Radius and radiale

**Fig 5:**
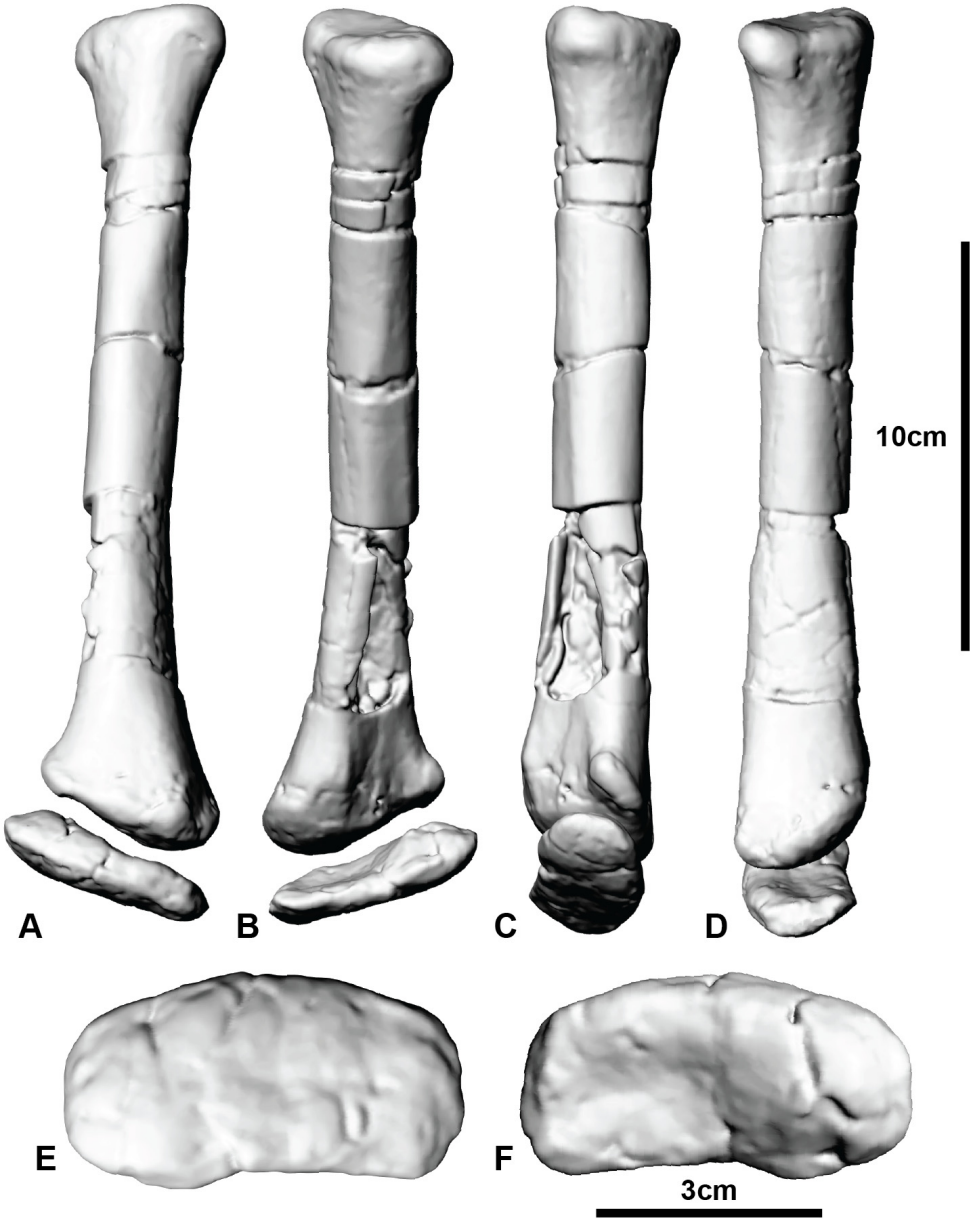
Articulated radius and radiale. Radius and radiale in: (A) cranial view; (B) caudal view; (C) lateral view; (D) medial view; (E) radiale in distal view; (F) radiale in proximal view.

The articulation of the antebrachium to the wrist is difficult to identify as the lateral margin of distal carpal 1 is not preserved. The radiale is complete and forms a slightly rounded cap for the radius (Fig 5; S16 Fig). The morphology of the radiale is important, as it dictates the articulation with the wrist and is a critical component of the wing folding mechanism in maniraptoran theropods [39]. Unlike maniraptorans, however, the radiale of *Australovenator* is proximodistally flattened with a weakly domed distal surface and lacks a distinct angle or facet that articulates well with distal carpal 1. This configuration, present in both *Allosaurus* and *Coelophysis* Cope [42], [43] is considered a probable primitive tetanuran condition [44]. Similar radiale morphology is also found in the carcharodontosaurid *Acrocanthosaurus*. Senter and Robins [13] suggested additional cartilaginous padding was present on the radiale of *Acrocanthosaurus*, which furthermore prevents accurate ROM analysis. A cap of enthesial cartilage was likely present also on the radiale of *Australovenator* therefore the ROM of the wrist in *Australovenator* cannot be accurately determined.

### Metacarpus

**Fig 6:**
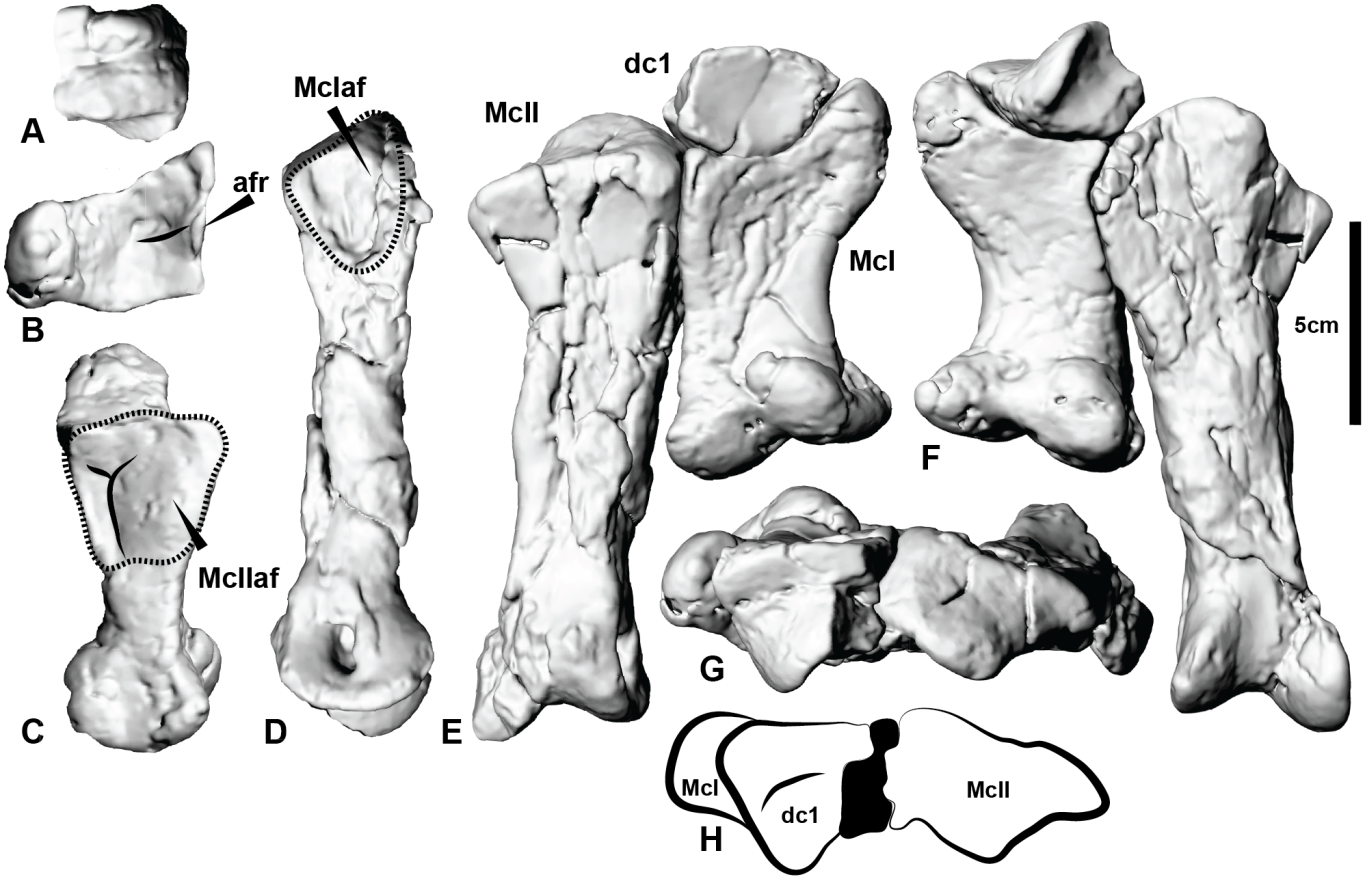
Distal carpal 1, metacarpal I and metacarpal II. (A) Distal carpal 1 in ventral view displaying the ventral articular facet that buttresses metacarpal I. (B) The proximal articular facet of metacarpal I where distal carpal I articulates. (C) The lateral face of metacarpal I displaying the articulating morphology with metacarpal II. (D) The medial face of metacarpal II displaying the articulating morphology of metacarpal. Articulated distal carpal I, metacarpal I and metacarpal II in: (E) Cranial view; (F) Ventral view; (G) proximal view; (H) proximal outline. Abbreviations: afr articular facet ridge; dc1 distal carpal 1, McI metacarpal I, McII metacarpal II, McIaf metacarpal I articular facet, McIIaf metacarpal II articular facet.

Metacarpal I and distal carpal 1 form a tightly-fitting joint that prohibited significant movement between the two elements. Together, these elements form a rounded fossa on their combined lateral surface that articulated with the proximomedial portion of metacarpal II. The proximal articular surface of metacarpal II reveals its articulation with the missing lateral section of distal carpal 1. In lateral view, the proximal half of metacarpal I is trapezoidal with a pronounced longitudinal v-shaped groove on the ventral (palmar) half of this trapezoid. The ventrolateral margin of metacarpal I also forms a sharp ridge (Fig 6, S6 Fig), which is bowed slightly ventrally in lateral view to receive a portion of the proximomedial end of metacarpal II. Together, the v-shaped groove and ventrolateral ridge create a tightly-fitting union between metacarpals I and II. The medial depression of metacarpal II that receives the lateral margin of metacarpal I was slightly deformed during fossilisation so an exact articulation could not be established. Nevertheless, the presence of multiple interlocking features between distal carpal 1, metacarpal I and metacarpal II suggest these elements formed a solid immoveable unit, consistent with other theropods [45] (Fig 6; S17 Fig). The proximal articular surface of this combined unit permits the entire manus to adduct in a in amedio-ventral direction (see Fig 11 in White et al. [4]). *Australovenator* shares a similar morphology of distal carpal 1 with *Allosaurus*. It occupies a position above the contact between metacarpals I and II which is also the case in *Allosaurus*, *Acrocanthosaurus* and *Coelophysis*. Due to this articulation in *Coelophysis* Currie and Carpenter [39] recognised it as a primitive condition.

### Digits

The digits possess significant extension and flexion capabilities. Digit I was capable of 82° extension and 90° flexion relative to metacarpal I, which is accentuated by hyperextension (42° relative to phalanx I-2) and flexion (80° relative to phalanx I-2) of the ungual.

**Fig 7:**
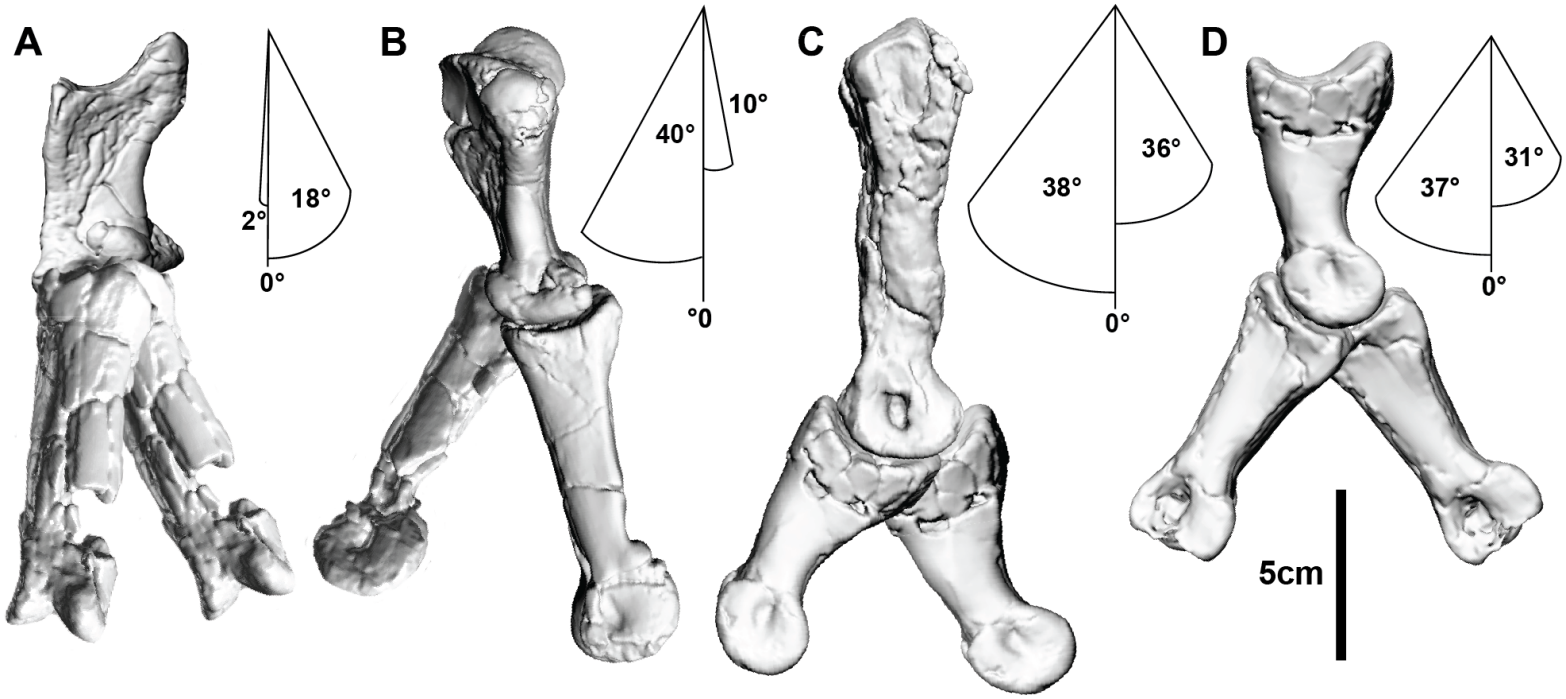
Phalangeal range of motion. (A) Cranial view of metacarpal I and manual phalanx I-1 displaying medial and lateral rotation. Extension and flexion in medial view of: (B) Metacarpal I and manual phalanx I-1; (C) Metacarpal II and McII-1; (D) Manual phalanx II-1 and II-2.

**Fig 8:**
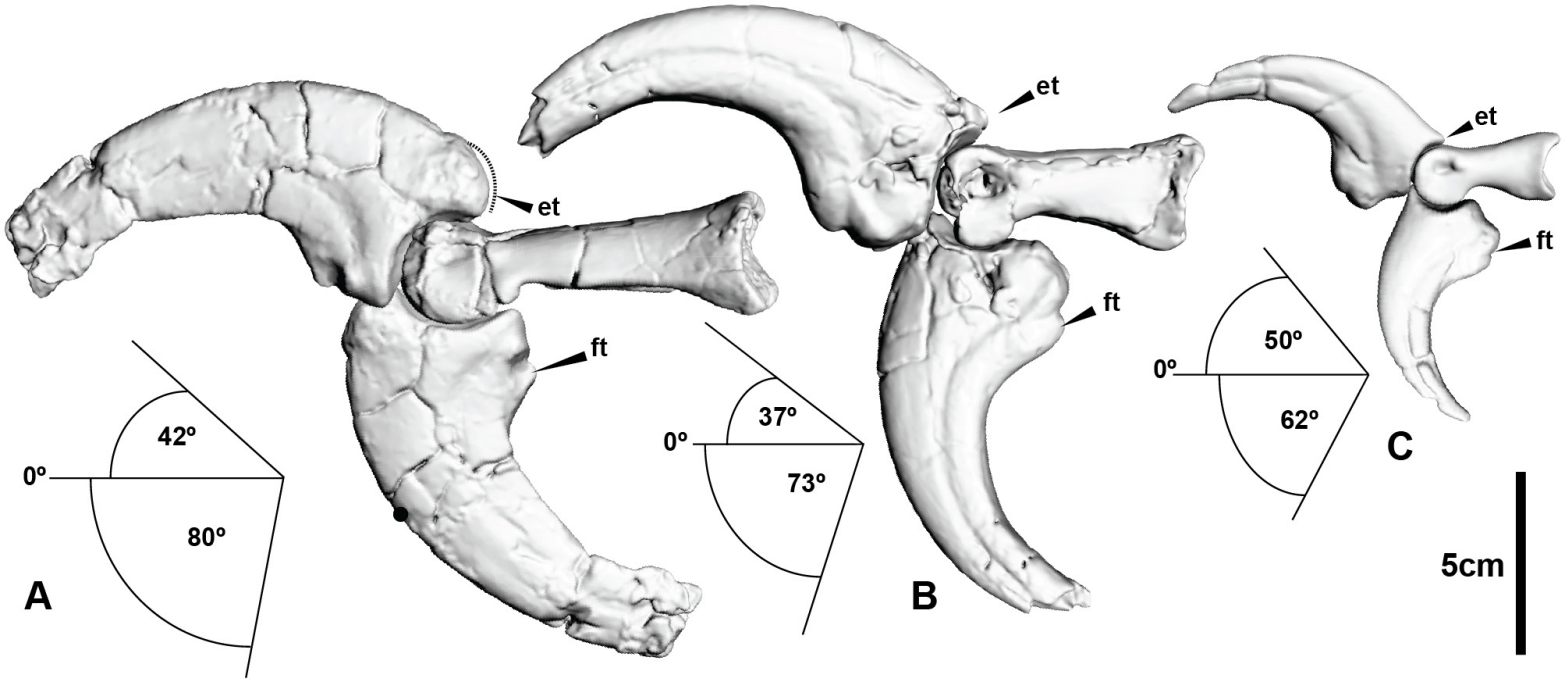
Manus ungual range of motion. (A) Manual phalanx I-2 extended and flexed. (B) Manual phalanx II-3 extend and flexed. (C) Manual phalanx III-4 extended and flexed.

Some mediolateral movement existed between metacarpal I and manual phalanx I-1 with a medial range of 18° and 2° in lateral movement. Digit II was capable of 112° of extension and 139° of flexion relative to metacarpal II. The ungual of digit II possessed the greatest ROM of any individual phalanx in the digit (73° and 37° of flexion and hyperextension, respectively). Digit III is only represented by manual phalanges III-3 and III-4, which exhibit a large ROM (50° and 62° hyperextension and flexion, respectively), similar to digits I and II (Table 1; Figs 7 and 8). During flexion digits 1 and 2 converge (S18 Fig).

**Table 1:** Range of motion (ROM) values of various theropods in comparison with *Australovenator*.

Dinosaur taxon		ANTEBRACHIUM	I-1	I-2	II-1	II-2	II-3	III-4	Information source
*Australovenator wintonensis*	*Extension*	144	40	42	38	37	37	50	**AODF604**
	*Flexion*	66	10	80	36	31	73	62	
	***ROM***	**78**	**50**	**122**	**72**	**68**	**110**	**112**	
*Allosaurus fragilis*	*Extension*	**?**	55		20	10	0		**Senter & Parrish (2005); Carpenter (2001)**
	*Flexion*	**?**	19		18	63	58		
	*ROM*	**62**	**74**		**38**	**73**	**58**		
*Acrocanthosaurus atokensis*	*Extension*	159	90	0	77	97	0	0	**Senter & Robins (2005)**
	*Flexion*	104	35	3	36	70	35	108	
	*ROM*	**55**	**125**	**3**	**113**	**167**	**35**	**108**	
*Bambiraptor feinbergi*	*Extension*	127, 136	15	0	28	7	6		**Senter & Parrish (2005); Senter (2006)**
	*Flexion*	59, 55	51	76	50	70	92		
	*ROM*	**68, 81**	**66**	**76**	**78**	**77**	**98**		
*Deinonychus antirrhopus*	*Extension*	150	43	4	10	0	11	11	**Senter & Parrish (2005)**
	*Flexion*	51	49	70	51	75	85	74	
	*ROM*	**99**	**92**	**74**	**61**	**75**	**96**	**85**	
*Ornitholestes hermanni*	*Extension*	58	29	0			17		**Senter & Parrish (2005); Senter (2006)b**
	*Flexion*	37	52	85			100		
	*ROM*	**95**	**81**	**85**			**117**	**0**	
*Chirostenotes pergracilis*	*Extension*		5	7	24	25, 16	4		**Senter & Parrish (2005)**
	*Flexion*		51	62	41	58, 60	52		
	*ROM*		**56**	**69**	**65**	**83, 76**	**56**		
*Coelophysis bauri*	*Extension*		18	26	17	13	10		**Senter & Parrish (2005)**
	*Flexion*		54	40	48	60	50		
	*ROM*		**72**	**66**	**65**	**73**	**60**		
*Tyrannosaurus rex*	*Extension*		35	34					**Senter & Parrish (2005); Carpenter & Smith (2001)**
	*Flexion*		18	22					
	*ROM*	**45**	**53**	**56**					
*Harpymimus okaldnikovi*	*Extension*		28	0	19	14	0		**Senter & Parrish (2005); Kobayashi & Barsbold (2005)**
	*Flexion*		70	97	25	46	90		
	*ROM*		**98**	**97**	**44**	**60**	**90**		
*Dilophosaurus wetherilli*	*Extension*			42		26	9		**Senter & Parrish (2005); Welles (1984)**
	*Flexion*		40	68	46	70	53		
	*ROM*			**110**		**96**	**62**		
*Gallimimus sp*.	*Extension*		20	0	25	0	0		**Senter & Parrish (2005); Kobayashi & Barsbold (2005)**
	*Flexion*		33	96	27	33	90		
	*ROM*		**53**	**96**	**52**	**33**	**90**		

The recurved manual unguals of *Australovenator* were capable of significant extension, in particular, manual ungual II-3, which had hyperextension capabilities distinctly greater than in any other theropod [13]. Although the two preserved unguals (I-2 and II-3) are similar in size, manual phalanx I-2 is distinctly asymmetrical with a relatively flattened lateral side and a more convex medial side compared with the more symmetrical morphology of manual phalanx II-3 [4]. Manual phalanx also exhibits a greater ROM than manual phalanx II-3, which may be indicative of a different function. The basal theropod *Dilophosaurus* shares with *Australovenator* the large ROM of ungual I-2; however, the extension capability of ungual II-3 is considerably less than *Australovenator*.

The ungual morphologies of *Acrocanthosaurus* are distinctly different to *Australovenator* with their spear like morphology [13]. *Ornitholestes* and the dromaeosaurids *Bambiraptor* and *Deinonychus* all have manual unguals with limited extension capabilities; however, they share large flexion capabilities similar to *Australovenator*.

## Discussion

Although the phylogenetic placement of *Australovenator* and other magaraptorans is still ambiguous, comparisons between *Australovenator* and other theropods provide unique insights into the possible evolutionary sequence of forelimb ROM.

Elbow flexion in theropods shows a strong correlation with phylogeny [20]. For instance, *Ornitholestes* had the ability to flex the antebrachium beyond a right angle forming an acute angle between the forearm and the humerus [20]. More basal theropods (*Coelophysis*, *Herrerasaurus*, *Tyrannosaurus*, *Dilophosaurus*, *Allosaurus* and *Acrocanthosaurus*) on the other hand possessed a more restricted flexion capability; the antebrachium and humerus forming an obtuse angle even when the forearm was fully flexed [9, 13, 14, 20, 24, 25, 46]. Interestingly, the relatively large degree of forearm flexion in *Australovenator* (66°) is consistent with the maniraptoriform design. Such a large antebrachium ROM is consistent with the following; increased ability to draw items closer to the chest; drawing arms closer to the midline reducing drag during fast locomotion; assisted in increased rotational inertia during locomotion and thermoregulation [18, 20].

The ability of the radius to move distally relative of the ulna during flexion in *Australovenator* is peculiar among most non-avian theropods. In modern birds, this movement is effected by an enlarged radial condyle of the humerus, which displaces the radius during flexion causing it to slide distally with respect to the ulna. Such a movement in modern birds contributes to the wing folding mechanism. This novel arrangement appears to be replicated (convergently acquired) in *Australovenator*; however, rather than wing folding, we infer this configuration further accentuated the medial flexion of the wrist. Interestingly, in the derived coelurosaurs *Bambiraptor* and *Deinonychus* there is a lack of proximal and distal rolling surfaces between the radius and ulna. This indicates that there was no movement between these elements in these dinosaurs [18] which further supports this is a homoplastic feature in *Australovenator*. The articulation of distal carpal 1 with metacarpal I and II is regarded as a primitive arrangement shared with *Coelophysis*, *Acrocanthosaurus* and *Allosaurus*.

The digits of *Australovenator* exhibit considerable hyperextension, which is a primitive feature, common to Allosauroidea and also *Dilophosaurus*. In Coelurosauria there is a distinct reduction of manual ungual hyperextensitivity, which is evident in *Bambiraptor*, *Deinonychus*, *Ornitholestes*, *Chirostenotes*, *Harpymimus*, *and Gallimimus*.

**Fig 9:**
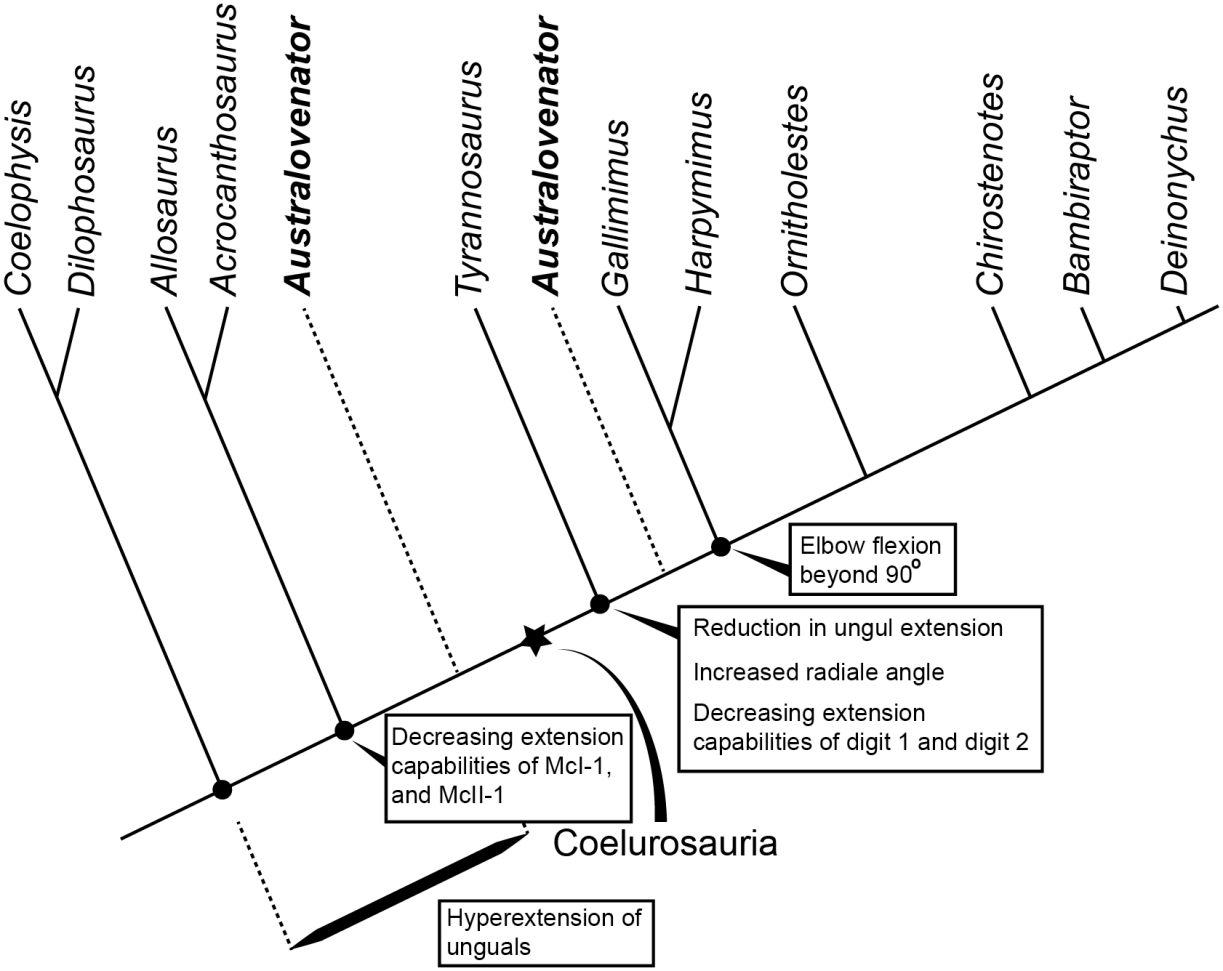
Phylogeny of theropods mentioned in the text. Various forearm range of motion evolutionary trends are identified. The phylogeny was created with reference to other range of motion analysis and more recent phylogenetic analysis [6, 13, 15, 20, 49].

*Australovenator* therefore displays a suite of both primitive and derived states with regards to forelimb ROM. Subsequently, it still remains inconclusive as to whether *Australovenator* belongs to a highly derived tetanuran group within Allosauroidea [1, 47–49] or was a basal coelurosaur [6, 7] (Fig 9).

The forearm ROM comparisons of *Australovenator* with other theropod groups provide insight into the functional role the forelimb played during prey capture and dispatch. Larger theropods such as tyrannosaurids and carcharodontosaurids possessed limited anterior reach suggesting that initial prey capture was made orally with the forearms used to maintain grip on prey [13]. The forearm lateral extension mobility of *Australovenator* possibly represented a predatory action that facilitated prey capture. The reasonably large ROM of the antebrachium indicates a large forearm extension and infers the capability to draw in the arms (and prey) close to the chest for easier dispatch. *Australovenator* had a gracile dentary and most likely possessed a relatively weak bite as a consequence. However, like tyrannosaurids, the unguals of digits I and II converged during flexion, which accentuated grip in an action of drawing the arms closer to the chest [24, 25, 50]. There is also some minor mediolateral movement achieved between the proximal phalanx of digit I and metacarpal I. This movement was relatively restricted compared to the dromaeosaurid *Bambiraptor*, which ostensibly possessed an opposable digit I [18]. As a result, *Australovenator* most likely required both hands to secure prey. Theropods with relatively weak bites relied on their hands and feet to assist in the dispatch of prey; however, the approach varied depending on the ROM and morphology of the forearms.

*Australovenator* appears to be closer in manual ROM to *Dilophosaurus* than to any other theropod, whereby the first digit hypothetically played a dominant role in prey dispatch [14]. Smaller theropods such as dromaeosaurids possessed greater manual flexion but lacked the extension capabilities of *Australovenator*. This emphasizes that the main function of the dromaeosaurid manus was to grapple, with dispatch achieved orally and/or with the sickle-like claw on its second pedal digit [51]. Hyperextension of the manual unguals of *Australoventor* is greater than in any other theropod compared. Subsequently this infers that *Australovenator* and megaraptorids alike possessed a unique manus function most likely associated with predation.

## Conclusion

The forearms of *Australovenator* are envisioned to have played a dominant role in prey capture. The morphology of the proximal end of the humerus reveals the forearms were capable of being extended to a sub-horizontal position but probably could not retract beyond a sub-vertical position. The unusual distal movement of the radius during flexion is suggested to have forced a medial flexion of the wrist. This movement would have accentuated the ability of the manus to draw food items closer to its chest. Distal carpal I and metacarpals I and II form an inflexible unit that is identified as a primitive configuration among theropods. Digits one and two converge slightly during flexion and expand when extended. The first ungual was blade like and had extreme hyperextension and flexion capabilities. The ungual on the second digit had slightly reduced hyperextension and flexion capabilities compared phalanx I-2. The ability to hyperextend the manual unguals and digits is widespread among basal tetanurans whereas the ability to flex the antebrachium beyond 90° was identified as a more derived coelurosaurian trait.

Considering all aspects of ROM, articulation and forearm morphology we conclude that *Australovenator* belongs to a highly derived tetanuran or a very basal coelurosaur.

## Supporting Information

S1 Fig Humerus. *Australovenator* specimens were discovered by and are housed in The Australian Age of Dinosaurs Museum of Natural History. Access to the specimens was granted by founder and chairman David A. Elliott. Computed tomography (CT) scanning: Sarah J. Wooldridge (Queensland Xray Mackay). Model reconstruction: Matt A. White (University of Newcastle). Visualisation David G. Barnes (Monash University).
(PDF)

S2 Fig Ulna. *Australovenator* specimens were discovered by and are housed in The Australian Age of Dinosaurs Museum of Natural History. Access to the specimens was granted by founder and chairman David A. Elliott. Computed tomography (CT) scanning: Sarah J. Wooldridge (Queensland Xray Mackay). Model reconstruction: Matt A. White (University of Newcastle). Visualisation David G. Barnes (Monash University).
(PDF)

S3 Fig Radius. *Australovenator* specimens were discovered by and are housed in The Australian Age of Dinosaurs Museum of Natural History. Access to the specimens was granted by founder and chairman David A. Elliott. Computed tomography (CT) scanning: Sarah J. Wooldridge (Queensland Xray Mackay). Model reconstruction: Matt A. White (University of Newcastle). Visualisation David G. Barnes (Monash University).
(PDF)

S4 Fig Radiale. *Australovenator* specimens were discovered by and are housed in The Australian Age of Dinosaurs Museum of Natural History. Access to the specimens was granted by founder and chairman David A. Elliott. Computed tomography (CT) scanning: Sarah J. Wooldridge (Queensland Xray Mackay). Model reconstruction: Matt A. White (University of Newcastle). Visualisation David G. Barnes (Monash University).
(PDF)

S5 Fig Distal carpal 1. *Australovenator* specimens were discovered by and are housed in The Australian Age of Dinosaurs Museum of Natural History. Access to the specimens was granted by founder and chairman David A. Elliott. Computed tomography (CT) scanning: Sarah J. Wooldridge (Queensland Xray Mackay). Model reconstruction: Matt A. White (University of Newcastle). Visualisation David G. Barnes (Monash University).
(PDF)

S6 Fig Metacarpal 1. *Australovenator* specimens were discovered by and are housed in The Australian Age of Dinosaurs Museum of Natural History. Access to the specimens was granted by founder and chairman David A. Elliott. Computed tomography (CT) scanning: Sarah J. Wooldridge (Queensland Xray Mackay). Model reconstruction: Matt A. White (University of Newcastle). Visualisation David G. Barnes (Monash University).
(PDF)

S7 Fig Manual phalanx I-1. *Australovenator* specimens were discovered by and are housed in The Australian Age of Dinosaurs Museum of Natural History. Access to the specimens was granted by founder and chairman David A. Elliott. Computed tomography (CT) scanning: Sarah J. Wooldridge (Queensland Xray Mackay). Model reconstruction: Matt A. White (University of Newcastle). Visualisation David G. Barnes (Monash University).
(PDF)

S8 Fig Manual phalanx I-2. *Australovenator* specimens were discovered by and are housed in The Australian Age of Dinosaurs Museum of Natural History. Access to the specimens was granted by founder and chairman David A. Elliott. Computed tomography (CT) scanning: Sarah J. Wooldridge (Queensland Xray Mackay). Model reconstruction: Matt A. White (University of Newcastle). Visualisation David G. Barnes (Monash University).
(PDF)

S9 Fig Metacarpal II. *Australovenator* specimens were discovered by and are housed in The Australian Age of Dinosaurs Museum of Natural History. Access to the specimens was granted by founder and chairman David A. Elliott. Computed tomography (CT) scanning: Sarah J. Wooldridge (Queensland Xray Mackay). Model reconstruction: Matt A. White (University of Newcastle). Visualisation David G. Barnes (Monash University).
(PDF)

S10 Fig Manual phalanx II-1. *Australovenator* specimens were discovered by and are housed in The Australian Age of Dinosaurs Museum of Natural History. Access to the specimens was granted by founder and chairman David A. Elliott. Computed tomography (CT) scanning: Sarah J. Wooldridge (Queensland Xray Mackay). Model reconstruction: Matt A. White (University of Newcastle). Visualisation David G. Barnes (Monash University).
(PDF)

S11 Fig Manual phalanx II-2. *Australovenator* specimens were discovered by and are housed in The Australian Age of Dinosaurs Museum of Natural History. Access to the specimens was granted by founder and chairman David A. Elliott. Computed tomography (CT) scanning: Sarah J. Wooldridge (Queensland Xray Mackay). Model reconstruction: Matt A. White (University of Newcastle). Visualisation David G. Barnes (Monash University).
(PDF)

S12 Fig Manual phalanx II-3. *Australovenator* specimens were discovered by and are housed in The Australian Age of Dinosaurs Museum of Natural History. Access to the specimens was granted by founder and chairman David A. Elliott. Computed tomography (CT) scanning: Sarah J. Wooldridge (Queensland Xray Mackay). Model reconstruction: Matt A. White (University of Newcastle). Visualisation David G. Barnes (Monash University).
(PDF)

S13 Fig Manual phalanx III-3. *Australovenator* specimens were discovered by and are housed in The Australian Age of Dinosaurs Museum of Natural History. Access to the specimens was granted by founder and chairman David A. Elliott. Computed tomography (CT) scanning: Sarah J. Wooldridge (Queensland Xray Mackay). Model reconstruction: Matt A. White (University of Newcastle). Visualisation David G. Barnes (Monash University).
(PDF)

S14 Fig Manual phalanx III-4. *Australovenator* specimens were discovered by and are housed in The Australian Age of Dinosaurs Museum of Natural History. Access to the specimens was granted by founder and chairman David A. Elliott. Computed tomography (CT) scanning: Sarah J. Wooldridge (Queensland Xray Mackay). Model reconstruction: Matt A. White (University of Newcastle). Visualisation David G. Barnes (Monash University).
(PDF)

S15 Fig Range of motion of humerus and ulna. *Australovenator* specimens were discovered by and are housed in The Australian Age of Dinosaurs Museum of Natural History. Access to the specimens was granted by founder and chairman David A. Elliott. Computed tomography (CT) scanning: Sarah J. Wooldridge (Queensland Xray Mackay). Model reconstruction: Matt A. White (University of Newcastle). Visualisation David G. Barnes (Monash University).
(PDF)

S16 Fig Radius and radiale in articulation. *Australovenator* specimens were discovered by and are housed in The Australian Age of Dinosaurs Museum of Natural History. Access to the specimens was granted by founder and chairman David A. Elliott. Computed tomography (CT) scanning: Sarah J. Wooldridge (Queensland Xray Mackay). Model reconstruction: Matt A. White (University of Newcastle). Visualisation David G. Barnes (Monash University).
(PDF)

S17 Fig Articulated distal carpal 1, metacarpal I, metacarpal II. *Australovenator* specimens were discovered by and are housed in The Australian Age of Dinosaurs Museum of Natural History. Access to the specimens was granted by founder and chairman David A. Elliott. Computed tomography (CT) scanning: Sarah J. Wooldridge (Queensland Xray Mackay). Model reconstruction: Matt A. White (University of Newcastle). Visualisation David G. Barnes (Monash University).
(PDF)

S18 Fig Range of motion of the manus. *Australovenator* specimens were discovered by and are housed in The Australian Age of Dinosaurs Museum of Natural History. Access to the specimens was granted by founder and chairman David A. Elliott. Computed tomography (CT) scanning: Sarah J. Wooldridge (Queensland Xray Mackay). Model reconstruction: Matt A. White (University of Newcastle). Visualisation David G. Barnes (Monash University).
(PDF)
