# Supplementary material for: Forearm Range of Motion in Australovenator wintonensis (Theropoda, Megaraptoridae)
Source: PLoS One. 2015 Sep 14;10(9):e0137709. doi: 10.1371/journal.pone.0137709 (PMC4569425; doi:10.1371/journal.pone.0137709)
Supplement: S16 Fig — Australovenator specimens were discovered by and are housed in The Australian Age of Dinosaurs Museum of Natural History. Access to the specimens was granted by founder and chairman David A. Elliott. Computed tomography (CT) scanning: Sarah J. Wooldridge (Queensland Xray Mackay). Model reconstruction: Matt A. White (University of Newcastle). Visualisation David G. Barnes (Monash University). (PDF) [file pone.0137709.s016.pdf]

Australovenator wintonensis Supplementary Figure 16 Right Radius and radiale articulation

Australovenator specimens were discovered by and are housed in The Australian Age of Dinosaurs Museum of Natural History. Access to the specimens was granted by founder and chairman David A. Elliott. Computed tomography (CT) scanning: Sarah J. Wooldridge (Queensland Xray Mackay). Model construction: Matt A. White (University of Newcastle). Digital articulation: Travis R. Tischler (AAOD). Visualisation: David G. Barnes (Monash University).
